# Quantitative assessment of CBCT image quality metrics and their translation to clinical Art workflow performance in breast cancer patients

**DOI:** 10.1002/acm2.70648

**Published:** 2026-06-07

**Authors:** Sunhwa Kim, Young Kyu Lee, Wonjoong Cheon, Yunji Seol, Byung‐Ock Choi, Young Nam Kang

**Affiliations:** ^1^ Department of Medical Sciences College of Medicine The Catholic University of Korea Seoul Republic of Korea; ^2^ Department of Radiation Oncology College of Medicine Seoul St. Mary's Hospital The Catholic University of Korea Seoul Republic of Korea; ^3^ Department of Proton Center National Cancer Center of Korea Goyang‐si Gyeonggi‐do Republic of Korea

**Keywords:** adaptive radiotherapy, breast cancer, CBCT image quality, contour agreement, dosimetric accuracy

## Abstract

**Background:**

Online adaptive radiotherapy (ART) relies on cone‐beam computed tomography (CBCT) imaging for daily treatment adaptation, but CBCT's physical limitations can compromise contour delineation and dose calculation accuracy. The relationship between image quality metrics and clinical ART workflow accuracy remains unclear.

**Purpose:**

To investigate the clinical impact of CBCT image quality on ART workflow accuracy in breast cancer patients by comparing contour agreement and dose calculation accuracy between Halcyon and TrueBeam systems, establishing how quantitative image quality metrics affect contour agreement and dose calculation accuracy within the ART workflow.

**Methods:**

Forty breast cancer patients treated with VMAT were equally divided between Halcyon (*n* = 20) and TrueBeam (*n* = 20). Phantom‐based image quality assessment evaluated HU uniformity, contrast‐to‐noise ratio (CNR), signal‐to‐noise ratio (SNR), and modulation transfer function (MTF) using Catphan 504. Planning CT and first‐day CBCT images were aligned using deformable registration. AI auto‐segmentation generated contours for six structures, evaluated using Dice Similarity Coefficient (DSC) and other geometric metrics. VMAT plans were recalculated on CBCT images using system‐specific HU‐to‐electron density calibration curves for dose‐volume histogram, 3D gamma analysis, and dose difference analysis.

**Results:**

Halcyon demonstrated 1.65‐fold higher CNR (22.21 vs. 13.42) and 1.78‐fold better HU uniformity (9 vs. 16 HU) compared to TrueBeam, despite comparable spatial resolution (MTF50: 0.36 lp/mm). Contour agreement analysis showed Halcyon achieved higher accuracy (treated breast DSC: 0.92 ± 0.06 vs. 0.87 ± 0.21, *p* < 0.001) with 3.5‐fold lower inter‐patient variability. Dosimetric analysis revealed paradoxical patterns: TrueBeam maintained better PTV coverage (D_95%_ difference: 0.013 vs. 0.207 Gy) but showed significantly increased internal dose metrics (D_mean_ difference: −0.320 vs. 0.046 Gy, *p* < 0.001). Although both systems achieved > 99.8% gamma passing rates at 3%/3 mm criteria, stringent 1%/2 mm criteria revealed substantial performance differences (Halcyon: 97.66 ± 1.91% vs. TrueBeam: 90.28 ± 6.51%, *p* < 0.001).

**Conclusion:**

Quantitative CBCT image quality metrics, particularly CNR and HU uniformity, directly affect both contour agreement and dosimetric concordance in breast ART workflows. These findings provide quantitative evidence linking CBCT image quality metrics to both contour agreement and dose calculation accuracy within the ART workflow.

## 1 INTRODUCTION

The introduction of cone‐beam computed tomography (CBCT) has established image‐guided radiotherapy (IGRT) as the standard of modern radiation therapy by enabling volumetric imaging of patients immediately before treatment.^1^, ^2^, ^3^ Unlike conventional approaches that relied on two‐dimensional (2D) portal imaging or external skin markers, CBCT‐based IGRT enables three‐dimensional (3D) volumetric visualization of patient anatomy, ensuring accurate localization of bony structures and permitting assessment of soft tissues.^1^, ^2^, ^4^, ^5^, ^6^, ^7^ This capability helps reduce setup errors and facilitates accurate dose delivery through image registration with the planning computed tomography (pCT) at each treatment fraction.^8^, ^9^, ^10^, ^11^, ^12^, ^13^

However, IGRT has inherent limitations, as it primarily focuses on minimizing setup errors without actively adapting to anatomical changes that occur during a treatment course.^14^, ^15^ Inter‐fraction variations such as patient weight loss, tumor volume changes, and positional shifts of adjacent organs at risk (OARs) can significantly compromise the intended dose distribution.^16^, ^17^, ^18^, ^19^ Although clinical practice attempts to address these uncertainties through target margin expansion, fixed margins alone are insufficient and may lead to target underdosing or unnecessary OAR irradiation.^20^, ^21^, ^22^ Therefore, adaptive radiotherapy (ART) has emerged to not only verify patient position and detect anatomical changes but also actively modify treatment plans based on the patient's current anatomical state.^23^, ^24^

ART can be implemented through different strategies: offline adaptation using replanning on newly acquired pCT between fractions, or online adaptation using CBCT images obtained during a treatment.^14^, ^25^ Although offline ART benefits from the superior image quality of pCT, the temporal delay between image acquisition and treatment delivery limits its effectiveness for rapidly changing anatomy. Online ART provides immediate adaptation capabilities through daily CBCT imaging; however, CBCT's physical limitations—including scatter, noise, artifacts, and restricted field of view—can adversely affect image quality metrics (geometric accuracy and Hounsfield Unit consistency), which in turn affects the clinical tasks of target and OAR delineation and dose calculation, making robust assessment of CBCT performance critical for successful online ART implementation.^17^, ^26^, ^27^, ^28^, ^29^, ^30^, ^31^, ^32^

Recent studies have systematically evaluated CBCT performance through phantom‐based assessments of quantitative image quality metrics including Hounsfield Unit (HU) accuracy and uniformity, contrast‐to‐noise ratio (CNR), modulation transfer function (MTF), and noise power spectrum (NPS), with further exploration of CBCT‐based online ART applicability through cross‐system comparisons.^33^, ^34^, ^35^, ^36^, ^37^ However, while these phantom‐based evaluations provide standardized performance benchmarks, the relationship between these quantitative metrics and actual contour agreement and dose calculation accuracy in patient data has not been thoroughly established.^38^, ^39^

**FIGURE 1:**
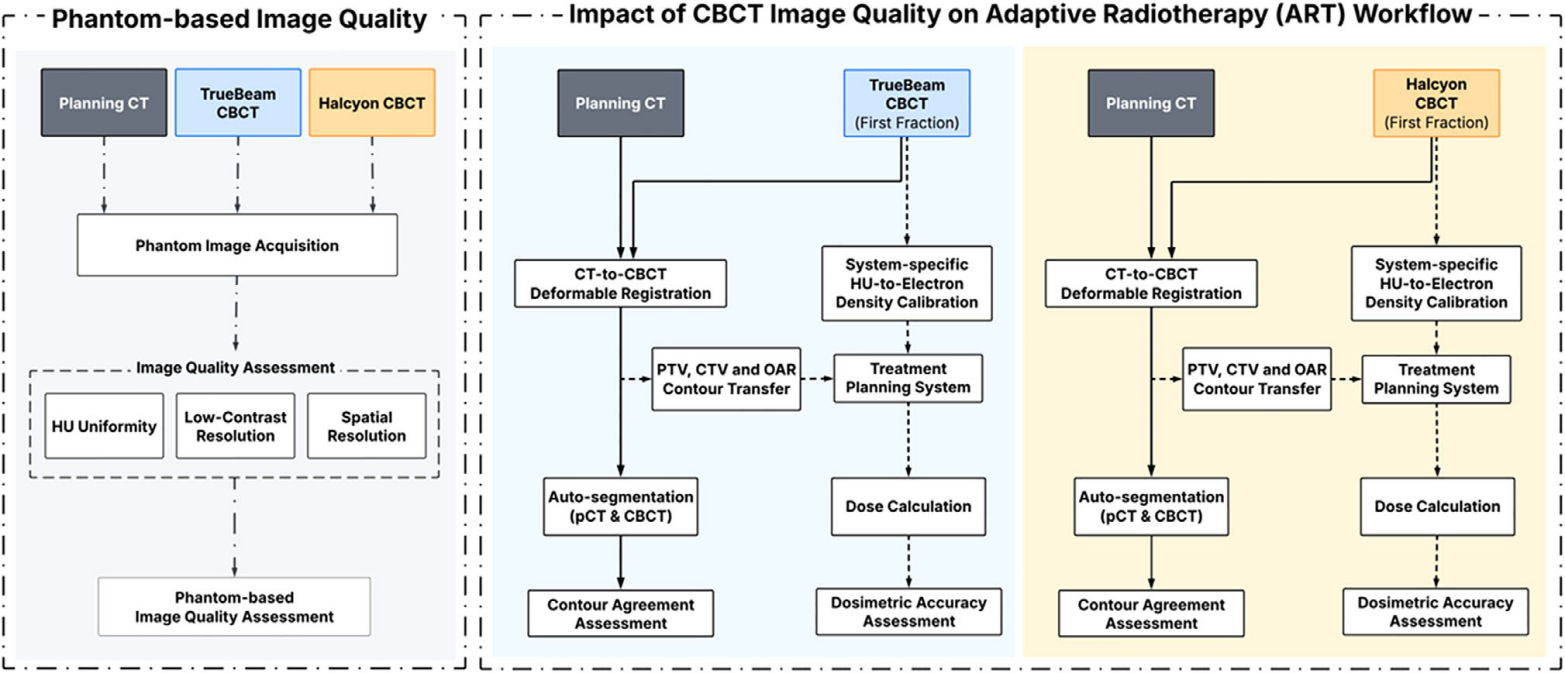
A flowchart illustrating the overall study workflow, from patient image acquisition to contour agreement and dosimetric analysis.

To address this knowledge gap, this study investigated the clinical impact of CBCT image quality on ART workflow in breast cancer patients. We evaluated anatomical segmentation consistency and dose calculation accuracy, comparing CBCT performance between Halcyon 3.1 and TrueBeam 3.0 systems (Varian Medical Systems, Palo Alto, CA, USA) to establish how image quality metrics affect contour agreement and dosimetric accuracy in the clinical ART workflow (Figure 1).

## 2 MATERIALS AND METHODS

### 2.1 Patient cohort and image acquisition

This retrospective study included a cohort of 40 breast cancer patients who had been treated with volumetric modulated arc therapy (VMAT). The cohort was equally divided between two linear accelerator systems: Halcyon (*n* = 20) and TrueBeam (*n* = 20). This study received approval from the Institutional Review Board (IRB), and all patient data were fully anonymized prior to analysis.

pCT scans were acquired using a SOMATOM go.Sim CT simulator (Siemens Healthineers, Erlangen, Germany) approximately 1 week prior to treatment initiation, with CBCT obtained during the first treatment fraction. All patients were imaged under free breathing conditions for both pCT simulation and CBCT acquisition, with no respiratory motion management techniques employed. In the Halcyon cohort, laterality was equally distributed (left: *n* = 10, 50%; right: *n* = 10, 50%), with 85% (*n* = 17) receiving breast‐only irradiation and 15% (*n* = 3) receiving breast and axillary nodal irradiation. The TrueBeam cohort demonstrated left‐sided predominance (left: *n* = 16, 80%; right: *n* = 4, 20%), with 55% (*n* = 11) receiving breast‐only treatment and 45% (*n* = 9) receiving breast and axillary nodal treatment. For patient immobilization, the Halcyon cohort was positioned supine with both arms raised using a dedicated breast board, while the TrueBeam cohort was positioned supine with both arms raised on a delta couch without a dedicated breast board.

All imaging was performed using standardized clinical protocols. pCT images were acquired at 120 kVp with 2.0 mm slice thickness. Detailed CBCT system configurations, including linac and imaging software versions, gantry design, scan parameters, and reconstruction settings, are summarized in Table 1. For CBCT acquisition, the imaging isocenter was positioned at the treatment isocenter defined during treatment planning for both systems. Prior to each CBCT scan, patients were aligned to the approximate isocenter position using skin reference markers, and the CBCT was then acquired and registered to the pCT for precise image‐guided positional correction. Image‐guided corrections consisted of translational shifts (lateral, longitudinal, and vertical) for both systems; couch rotations were not applied in either cohort. The CBCT images used for all subsequent analyses were those obtained after this image‐guided alignment process.

**TABLE 1:** CBCT system technical specifications.

Parameter	Halcyon CBCT	TrueBeam CBCT
Linac Version	Halcyon 3.1	TrueBeam 3.0
Imaging Software Version	2.35.3.1	2.22.5.0
Flat Panel Detector	RTI 4343L	RTI 4030CB
Gantry Design	Ring‐gantry (O‐ring)	C‐arm
Source‐to‐Imager Distance	154 cm	150 cm
Scan Rotation	360° (CW)	360° (CC)
Bow‐tie Filter	Titanium V2, Half‐Fan V2	Titanium V1, Half‐Fan V1
Tube Voltage (kVp)	125	125
Tube Current (mA)	10	15
Exposure Time (s)	4.91	17.90
Cumulative mAs	49.1	268.5
CTDIvol (mGy)	0.90	4.00
Reconstruction Algorithm	FDK	FDK
Convolution Kernel	Not available	Ram‐Lak
Slice Thickness (mm)	2.0	2.0
Clinical Protocol	Breast	Thorax

Abbreviations: CC, counter‐clockwise; CW, clockwise; FDK, Feldkamp‐Davis‐Kress.

### 2.2 Phantom‐based image quality assessment

To determine how CBCT image quality parameters influence contour agreement and dosimetric concordance assessment in ART workflows, standardized phantom measurements were performed. A Catphan 504 phantom (The Phantom Laboratory, Salem, NY, USA) was scanned on each system using identical protocols as patient CBCT acquisitions. Image quality metrics were analyzed using DoseLab software, version 7.0 (Varian Medical Systems, Palo Alto, CA, USA).

For HU value uniformity assessment, regions of interest (ROIs) were drawn at the center, top, bottom, left, and right of homogeneous regions. Uniformity was calculated as the maximum deviation between the central ROI and peripheral ROIs.^40^ HU*_center_* and HU*_peripheral_* represent the mean HU values of the central ROI and each of the four peripheral ROIs (top, bottom, left, right), respectively. HU uniformity was quantified as shown in Equation (1):

(1) $$Uniformity=max\left|HU_{\mathrm{peripheral}}-HU_{\mathrm{center}}\right|,$$

Low‐contrast detectability was evaluated using Polystyrene and Acrylic inserts within the CTP404 module. Contrast‐to‐noise ratio (CNR) and signal‐to‐noise ratio (SNR) were calculated using Equation (2) and Equation (3), respectively:

(2) $$CNR=\left|HU_{p\mathrm{olystyrene}}-HU_{\mathrm{acrylic}}\right|\;/\sigma \_\mathrm{acry},$$

(3) $$SNR=(HU_{\mathrm{acrylic}}+1000)/\sigma \_\mathrm{acry},$$

where HU*_polystyrene_* and HU*_acrylic_* represent the mean HU values within the Polystyrene and Acrylic ROIs, respectively, and σ_acry is the standard deviation (SD) of the HU values within the Acrylic ROI.^40^

Spatial resolution was evaluated using the CTP528 module of the Catphan phantom. Modulation transfer function (MTF) values at 50% and 10% modulation (MTF50 and MTF10) were determined using the automated algorithm in DoseLab software.

### 2.3 Contour agreement assessment

This evaluation was designed to assess the impact of image quality differences across imaging modalities (pCT, TrueBeam CBCT, and Halcyon CBCT) on anatomical delineation using an AI‐based auto‐segmentation algorithm, Contour+ (MVision AI, Helsinki, Finland). The use of automated segmentation eliminates inter‐observer variability, allowing direct assessment of how image quality affects segmentation performance across different systems.

For each patient, the pCT was deformably registered to the corresponding first‐day CBCT using the intensity‐based free‐form deformable registration (FFD) algorithm implemented in MIM Maestro v7.3 (MIM Software Inc., Cleveland, OH, USA) to establish a consistent anatomical reference frame.^41^ All registrations were executed with the software's default parameter settings without manual intervention or user‐guided adjustments, ensuring consistency across all cases and eliminating inter‐user variability. The auto‐segmentation algorithm was first applied to the deformably registered pCT to generate reference contours, which served as the ground truth. The same algorithm was then applied independently to both Halcyon and TrueBeam CBCT images.

Six anatomical structures were evaluated as OARs: treated breast, heart, ipsilateral lung, contralateral lung, contralateral breast, and esophagus. Contour agreement between CBCT‐derived and reference deformed pCT contours was quantified using the following metrics: Dice Similarity Coefficient (DSC), Jaccard Index (JI), 95% Hausdorff Distance (HD95), Mean Surface Distance (MSD), and Volume Similarity (VS).

For each system, statistical differences between deformed pCT‐derived and CBCT‐derived contour agreement metrics were assessed using the Wilcoxon signed‐rank test, with *p*‐values < 0.05 considered statistically significant.

### 2.4 Dosimetric accuracy assessment

**FIGURE 2:**
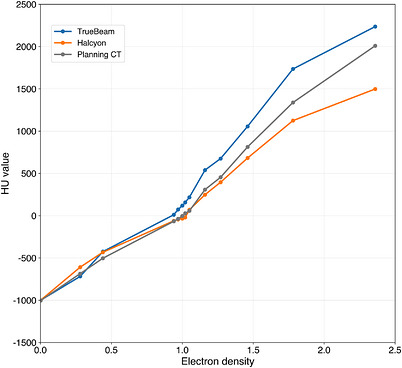
System‐specific HU‐to‐ED calibration curves for pCT, TrueBeam CBCT, and Halcyon CBCT.

System‐specific HU to electron density (ED) calibration curves were established for each imaging modality (pCT, TrueBeam CBCT, Halcyon CBCT) to ensure accurate dose calculation. The Advanced Electron Density Phantom (Sun Nuclear Corporation, Melbourne, FL, USA) was scanned on each system using identical acquisition parameters as patient imaging. HU values were measured across multiple electron density materials within the phantom to generate distinct calibration curves. These system‐specific curves were subsequently imported into the Eclipse Treatment Planning System v16.1 (Varian Medical Systems, Palo Alto, CA, USA) for dose calculation using the Acuros XB algorithm (Figure 2, Table A1).

To independently evaluate the dosimetric impact of CBCT image quality, the reference contours (CTV, PTV, and OARs) from the pCT were mapped onto the registered CBCT images via automated deformable contour registration in MIM Maestro v7.3. This approach ensured that any dosimetric differences would be attributed solely to image quality rather than contour discrepancies. The reference dose was calculated on the deformed pCT using the pCT‐specific HU‐to‐ED calibration curve. For each patient, the original VMAT treatment plan was recalculated on both Halcyon and TrueBeam CBCT using their respective HU‐to‐ED calibration curves, with all predefined plan parameters (including beam geometries, MUs, gantry angles, MLC sequences, and control points) kept constant. This within‐system comparison design ensures that the observed dosimetric differences are attributable to HU (electron density) differences rather than system‐specific delivery or contour‐related factors.

Dosimetric concordance was evaluated through both dose‐volume histogram (DVH) analysis for volumetric dose assessment and 3D gamma analysis for spatial dose distribution agreement.

For DVH analysis, target coverage metrics included D_95%_ (dose received by 95% of the volume) and V_100%_ (volume receiving 100% of the prescription dose), while internal dose distribution metrics comprised D_2%_ (near‐maximum dose) and D_mean_ (mean dose). For OARs, V_5Gy_ (volume receiving 5 Gy), D_mean_, and D_2%_ were evaluated. For each system, statistical differences between pCT‐based and CBCT‐based dose calculations were assessed using the Wilcoxon signed‐rank test, with *p* < 0.05 considered significant.

Spatial dose distribution agreement was quantified using 3D gamma analysis performed with MATLAB R2023b (MathWorks, Natick, MA, USA), comparing CBCT‐recalculated doses to deformed pCT‐based doses. Multiple gamma criteria were evaluated to assess dose agreement at different tolerance levels: 3%/3 mm, 2%/2 mm, 2%/1 mm, and 1%/2 mm (dose difference/distance‐to‐agreement), all with a 10% low‐dose threshold. Additionally, dose difference analysis was performed to directly quantify the magnitude of dosimetric discrepancies between pCT and CBCT‐recalculated dose distributions. Mean dose difference and mean absolute error (MAE) were calculated in both absolute (Gy) and relative terms using local normalization.

## 3 RESULTS

### 3.1 Image quality characteristics

#### 3.1.1 HU uniformity

**FIGURE 3:**
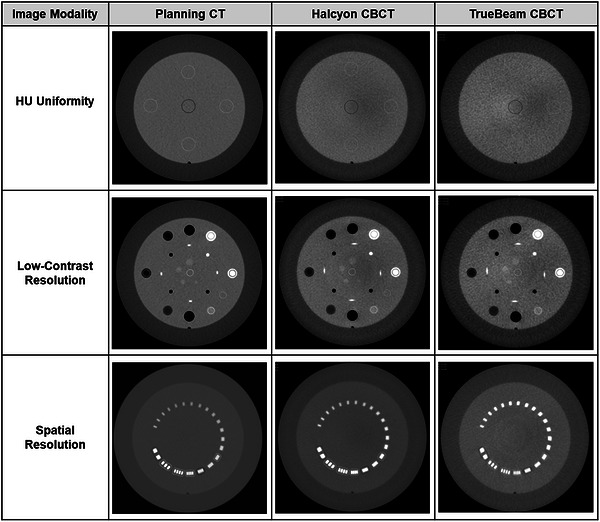
Catphan 504 phantom images demonstrating image quality metrics. Columns correspond to the three imaging modalities (left to right: Planning CT, Halcyon CBCT, and TrueBeam CBCT), and rows correspond to the assessment modules: HU uniformity (top), low‐contrast resolution (middle), and spatial resolution (bottom). Color‐coded ROIs indicate measurement locations for quantitative analysis.

The pCT, serving as the ground truth, demonstrated excellent uniformity at 2 HU. Halcyon and TrueBeam CBCT systems showed uniformity values of 9 HU and 16 HU, respectively. HU uniformity measurements for each imaging modality are summarized in Table 2 and Figure 3.

**TABLE 2:** Comparison of image quality characteristics across different imaging modalities.

Image modality	HU uniformity	CNR	SNR	MTF 50	MTF 20	MTF 10
Planning CT	2	26.14	185.57	0.37	0.55	0.67
Halcyon CBCT	9	22.21	164.16	0.36	0.51	N/A
TrueBeam CBCT	16	13.42	94.29	0.36	0.48	0.65

*Note*: HU uniformity, contrast‐to‐noise ratio (CNR), signal‐to‐noise ratio (SNR), and modulation transfer function values at 50% (MTF50), 20% (MTF20), and 10% (MTF10) were measured using Catphan 504 phantom for planning CT, Halcyon CBCT, and TrueBeam CBCT.

**TABLE 3:** Mean HU values and standard deviations for acrylic and polystyrene inserts across the three imaging modalities.

Image modality	Acrylic Mean HU	Acrylic SD	Polystyrene Mean HU	Polystyrene SD
**Planning CT**	122.70	6.05	−35.44	4.69
**Halcyon CBCT**	124.51	6.85	−27.60	6.44
**TrueBeam CBCT**	123.03	11.91	−36.81	13.38

*Note*: Mean HU values and standard deviations (SD) are reported for acrylic (nominal HU ≈ 120) and polystyrene (nominal HU ≈ −35) inserts within the CTP404 module across the three imaging modalities.

#### 3.1.2 Low‐contrast resolution

Low‐contrast resolution was evaluated using CNR and SNR measurements (Table 2, Figure 3), derived from the HU values and standard deviations of the acrylic and polystyrene inserts (Table 3). For CNR, pCT demonstrated the highest value at 26.14, followed by Halcyon CBCT at 22.21 and TrueBeam CBCT at 13.42. Halcyon CBCT showed approximately 1.65‐fold higher CNR compared to TrueBeam CBCT, with values closer to the pCT reference. Similarly, SNR measurements showed pCT with the highest value of 185.57, while Halcyon CBCT and TrueBeam CBCT recorded 164.16 and 94.29, respectively. Halcyon CBCT demonstrated approximately 1.74‐fold higher SNR than TrueBeam CBCT, confirming consistent performance differences between the two CBCT systems across both low‐contrast metrics.

#### 3.1.3 Spatial resolution

**FIGURE 4:**
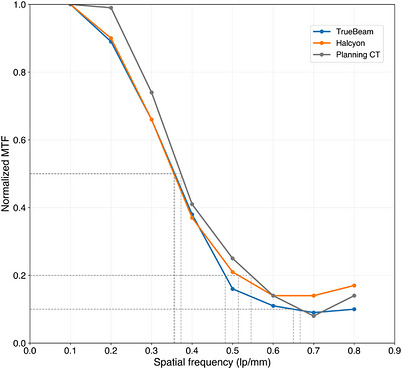
Normalized modulation transfer function (MTF) curves for the Planning CT, Halcyon CBCT, and TrueBeam CBCT.

Spatial resolution was evaluated using the MTF derived from the CTP528 module (Table 2, Figure 4). The MTF50 values were 0.37 lp/mm for pCT, and 0.36 lp/mm for both TrueBeam and Halcyon CBCT systems. MTF20 values demonstrated a gradual decrease from pCT (0.55 lp/mm) to Halcyon (0.51 lp/mm) and TrueBeam (0.48 lp/mm). MTF10 values were 0.67 lp/mm for pCT and 0.65 lp/mm for TrueBeam, while Halcyon's MTF10 could not be measured.

### 3.2 Contour agreement assessment

**FIGURE 5:**
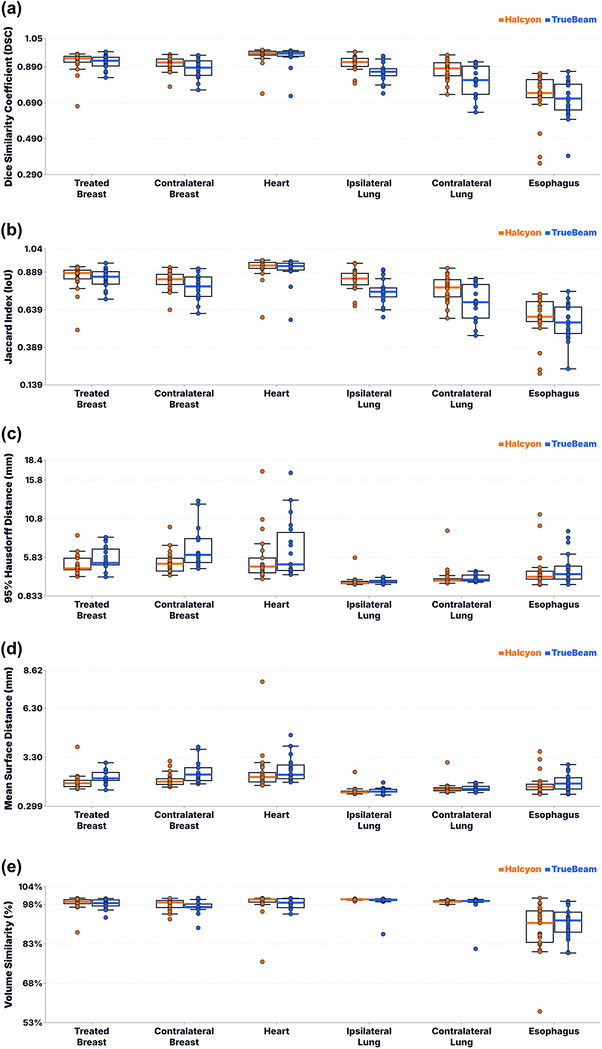
Contour agreement assessment of AI‐based auto‐segmentation on Halcyon (orange) and TrueBeam (blue) CBCT across six structures using five metrics (a) Dice Similarity Coefficient (DSC), (b) Jaccard Index (JI), (c) 95% Hausdorff Distance (HD95), (d) Mean Surface Distance (MSD), and (e) Volume Similarity (VS).

Contour agreement analysis between auto‐segmentations generated on CBCT images and those generated on deformably registered pCT demonstrated consistent superiority of Halcyon CBCT over TrueBeam CBCT across all evaluated structures (Figure 5). While both systems achieved clinically acceptable mean DSC values, substantial differences emerged in segmentation consistency. For the treated breast, Halcyon achieved a DSC of 0.92 ± 0.06 compared to TrueBeam's 0.87 ± 0.21, with the 3.5‐fold difference in SD indicating markedly higher inter‐patient variability for TrueBeam. This pattern of reduced consistency with TrueBeam persisted across most structures: contralateral breast (SD: ± 0.21 vs. ± 0.04), heart (± 0.22 vs. ± 0.05), and ipsilateral lung (± 0.20 vs. ± 0.04). Mean surface distances were consistently lower for Halcyon across all structures compared to TrueBeam. Detailed quantitative metrics are presented in Appendix Table A2.

To characterize the imaging properties underlying dose calculation, structure‐specific HU values, volumes, and relative electron densities were compared between the deformed pCT and CBCT for both systems (Tables 4, 5, 6).

**TABLE 4:** Structure HU values.

Structure	Halcyon deformed pCT (mean ± SD)	Halcyon CBCT (mean ± SD)	TrueBeam deformed pCT (mean ± SD)	TrueBeam CBCT (mean ± SD)
**Treated breast**	−65.84 ± 26.66	−70.48 ± 42.22	−68.94 ± 22.25	−93.60 ± 24.92
**Contralateral breast**	−72.93 ± 22.12	−85.50 ± 26.43	−73.10 ± 24.44	−96.27 ± 24.77
**Ipsilateral lung**	−724.30 ± 32.98	−748.42 ± 32.55	−701.82 ± 44.65	−730.67 ± 54.15
**Contralateral lung**	−723.45 ± 38.67	−739.02 ± 41.45	−715.72 ± 33.71	−735.36 ± 37.30
**Heart**	15.40 ± 7.15	2.35 ± 24.28	22.08 ± 15.46	14.62 ± 11.92

**TABLE 5:** Structure volume (cc).

Structure	Halcyon deformed pCT (mean ± SD)	Halcyon CBCT (mean ± SD)	TrueBeam deformed pCT (mean ± SD)	TrueBeam CBCT (mean ± SD)
**Treated breast**	298.82 ± 111.92	298.78 ± 111.93	392.61 ± 198.40	392.55 ± 198.37
**Contralateral breast**	308.25 ± 109.36	308.16 ± 109.40	355.63 ± 174.60	355.59 ± 174.61
**Ipsilateral lung**	1316.80 ± 195.24	1316.74 ± 195.27	1109.52 ± 196.32	1109.50 ± 196.36
**Contralateral lung**	1296.80 ± 226.19	1296.74 ± 226.21	1261.61 ± 264.24	1261.56 ± 264.23
**Heart**	544.57 ± 76.10	544.51 ± 76.04	559.74 ± 125.96	559.71 ± 125.96

**TABLE 6:** Electron density relative to water.

Structure	Halcyon deformed pCT (mean ± SD)	Halcyon CBCT (mean ± SD)	TrueBeam deformed pCT (mean ± SD)	TrueBeam CBCT (mean ± SD)
**Treated breast**	0.9392 ± 0.0274	0.9316 ± 0.0615	0.9363 ± 0.0237	0.8200 ± 0.0285
**Contralateral breast**	0.9320 ± 0.0242	0.9096 ± 0.0412	0.9317 ± 0.0264	0.8170 ± 0.0284
**Ipsilateral lung**	0.2460 ± 0.0289	0.2337 ± 0.0289	0.2657 ± 0.0392	0.2607 ± 0.0438
**Contralateral lung**	0.2468 ± 0.0339	0.2411 ± 0.0352	0.2535 ± 0.0296	0.2598 ± 0.0332
**Heart**	1.0117 ± 0.0046	1.0233 ± 0.0245	1.0164 ± 0.0112	0.9397 ± 0.0090

### 3.3 Dosimetric accuracy assessment

#### 3.3.1 Dosimetric assessment of target volumes

**FIGURE 6:**
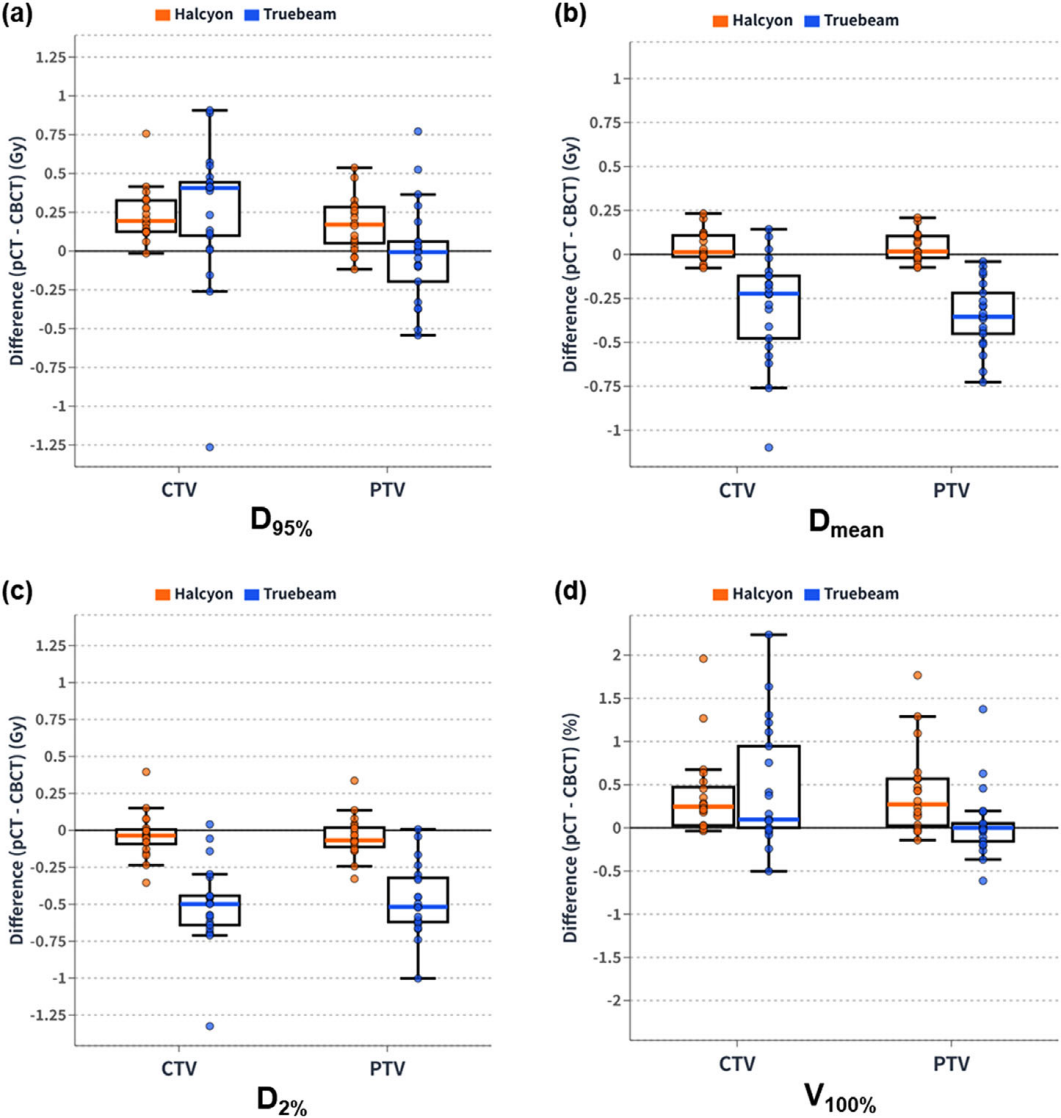
Target volume dosimetric differences (deformed pCT—CBCT) for (a) D_95%_, (b) D_mean_, (c) D_2%_, and (d) V_100%_.

To evaluate dosimetric accuracy, pCT contours (original targets and auto‐segmented OARs) were transferred to CBCT images via deformable registration. Forward dose calculations were then performed by applying the original VMAT plan to the deformed pCT and corresponding CBCT datasets using their respective system‐specific HU‐to‐ED calibration curves. The DVH differences (Δ = deformed pCT—CBCT) were analyzed (Figure 6). The analysis revealed divergent trends between target coverage metrics and internal dose distribution metrics.

For PTV coverage metrics (D_95%_, V_100%_), TrueBeam CBCT‐based calculations showed no statistically significant difference from the pCT reference (D_95%_: Δ = 0.013 Gy, *p* = 0.869; V_100%_: Δ = 0.087%, *p* = 0.486). In contrast, Halcyon CBCT showed reduced target coverage with statistical significance (D_95%_: Δ = 0.207 Gy, *p* < 0.001; V_100%_: Δ = 0.453%, *p* = 0.004).

For internal dose distribution metrics (D_mean_ and D_2%_), an inverse pattern emerged. TrueBeam CBCT showed significantly increased values for both metrics (D_mean_: Δ = −0.320 Gy, *p* < 0.001; D_2%_: Δ = −0.442 Gy, *p* < 0.001), while Halcyon CBCT showed small but statistically significant differences from the pCT reference (D_mean_: Δ = 0.046 Gy, *p* = 0.030; D_2%_: Δ = 0.084 Gy, *p* = 0.019).

#### 3.3.2 Dosimetric assessment of OARs

**FIGURE 7:**
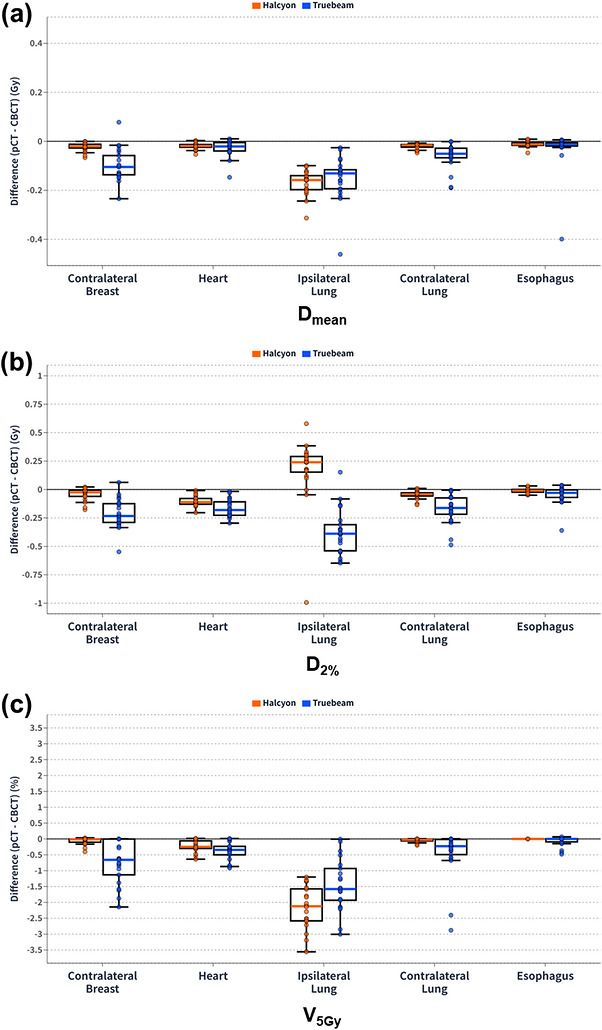
OARs dosimetric differences (deformed pCT—CBCT) for (a) D_mean_, (b) D_2%_ and (c) V_5Gy_.

To assess the dosimetric impact on OARs, the differences (Δ = deformed pCT—CBCT) between the pCT plan and the forward‐calculated CBCT doses were visualized (Figure 7).

TrueBeam CBCT demonstrated a consistent trend of more negative mean Δ values than Halcyon for the contralateral breast, heart, and contralateral lung. For example, the mean D_2%_ Δ for the contralateral breast was −0.215 Gy for TrueBeam (vs. −0.043 Gy for Halcyon), and for the heart, it was −0.166 Gy (vs. −0.102 Gy).

For ipsilateral lung, the dosimetric differences varied by metric. D_mean_ showed comparable increases between systems (Halcyon: Δ = −0.170 Gy, TrueBeam: Δ = −0.152 Gy, difference of 0.018 Gy). For V_5Gy_, Halcyon demonstrated 1.5‐fold greater increase (Δ = −2.159% vs. −1.458% for TrueBeam). Most notably, D_2%_ revealed opposing dosimetric biases: TrueBeam showed increased values of 0.375 Gy while Halcyon showed decreased values of 0.166 Gy, representing a 0.541 Gy difference in dose calculation.

#### 3.3.3 3D gamma analysis

**FIGURE 8:**
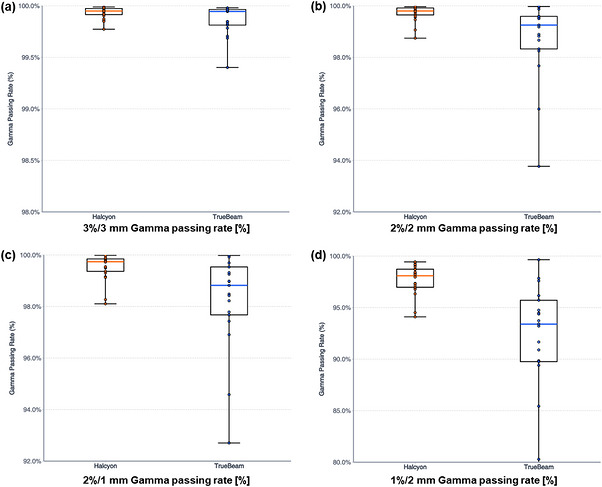
Three‐dimensional (3D) gamma passing rates for Halcyon and TrueBeam CBCT systems across various gamma criteria, comparing CBCT‐recalculated dose to deformed pCT dose. (a) Gamma criteria: 3% dose difference and 3.0 mm distance‐to‐agreement (DTA). (b) Gamma criteria: 2% dose difference and 2.0 mm DTA. (c) Gamma criteria: 2% dose difference and 1.0 mm DTA. (d) Gamma criteria: 1% dose difference and 2.0 mm DTA.

3D gamma analysis was performed to evaluate spatial dose distribution agreement between deformed pCT and CBCT‐recalculated dose distributions (Figure 8). Using the clinically standard 3%/3 mm criteria with a 10% dose threshold, both systems demonstrated excellent mean passing rates exceeding 99.8% (Halcyon: 99.93 ± 0.06%, TrueBeam: 99.87 ± 0.14%). However, when more stringent criteria were applied to detect low‐magnitude dose discrepancies arising from CBCT HU inaccuracies, substantial performance differences emerged between systems. Under the 1%/2 mm criteria, Halcyon CBCT maintained robust passing rates of 97.66 ± 1.91%, while TrueBeam CBCT showed significantly reduced performance with mean passing rates of 90.28 ± 6.51%.

**FIGURE 9:**
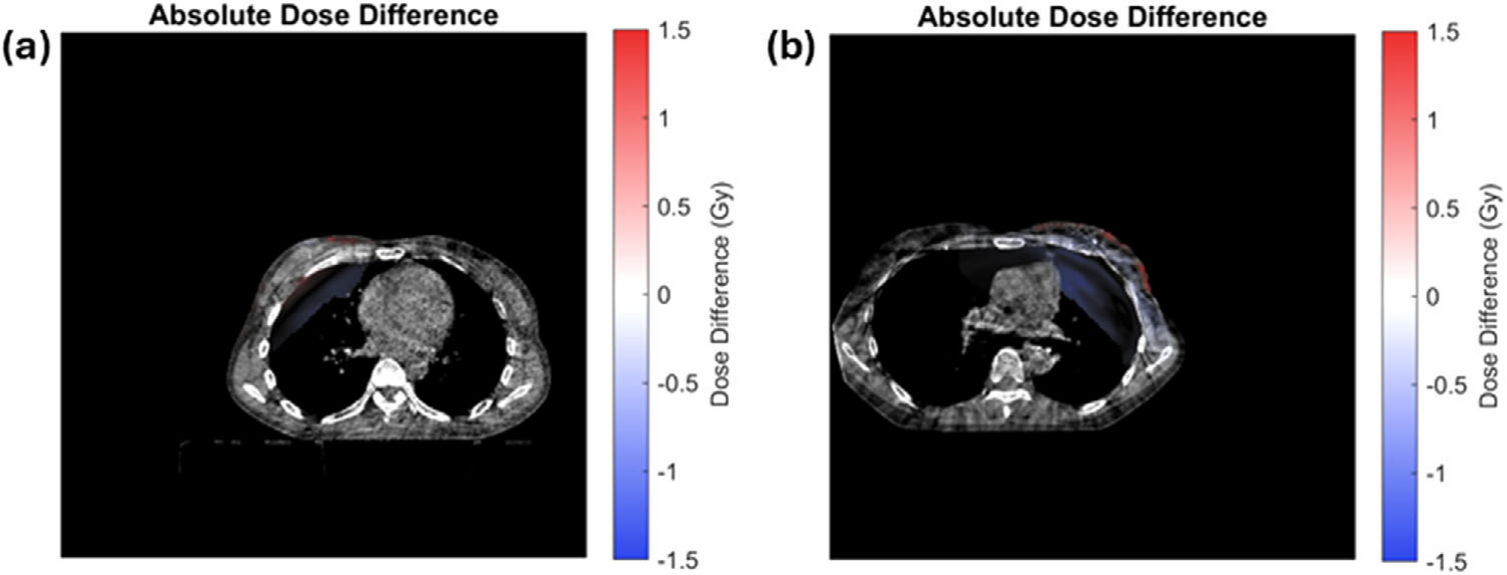
Representative axial absolute dose difference maps (Δ = deformed pCT − CBCT) for (a) Halcyon CBCT and (b) TrueBeam CBCT.

TrueBeam consistently demonstrated higher variability across all gamma criteria compared to Halcyon. Most notably, at the 1%/2 mm criteria, TrueBeam's SD was approximately 3.4‐fold higher than Halcyon's (6.51 %p vs. 1.91 %p), with individual patient passing rates for TrueBeam ranging from 78.3% to 98.5%.

Dose difference analysis results are summarized in Table 7, with representative dose difference maps shown in Figure 9. In the absolute analysis, Halcyon CBCT showed a mean dose difference of −0.05 ± 0.05 Gy with an MAE of 0.20 ± 0.07 Gy, while TrueBeam CBCT showed a mean dose difference of −0.26 ± 0.09 Gy with an MAE of 0.37 ± 0.08 Gy. In the relative local analysis, the mean dose difference was −0.84 ± 0.40% for Halcyon and −1.86 ± 0.47% for TrueBeam, with MAEs of 1.54 ± 0.61% and 2.36 ± 0.41%, respectively. These results indicate that Halcyon CBCT yields smaller systematic dose deviations compared to TrueBeam, consistent with the gamma analysis findings.

**TABLE 7:** Dose difference comparing deformed pCT and CBCT.

		Halcyon (mean ± SD)	TrueBeam (mean ± SD)
**Absolute (Gy)**	**Mean dose difference**	−0.05** ± **0.05	−0.26** ± **0.09
	**MAE**	0.20** ± **0.07	0.37** ± **0.08
**Relative, local (%)**	**Mean dose difference**	−0.84** ± **0.40	−1.86** ± **0.47
	**MAE**	1.54** ± **0.61	2.36** ± **0.41

## 4 DISCUSSION

This study provides quantitative evidence linking phantom‐based CBCT image quality metrics to both contour agreement and dose calculation accuracy within the ART workflow in breast cancer patients. While previous studies have separately evaluated either CBCT image quality through phantom experiments or dose calculation accuracy in isolation, they have not demonstrated how these quality differences translate into actual contour agreement and dose calculation accuracy in patient data.^42^, ^43^, ^44^ Our approach bridges this gap by systematically connecting phantom‐derived metrics with clinical patient outcomes across both domains. By employing identical AI algorithms for segmentation and consistent pCT‐based contours for dose calculation, we decoupled the direct impact of CBCT image quality from confounding factors such as inter‐observer variability.^45^ The systematic performance differences observed between Halcyon and TrueBeam systems across all evaluated domains—image quality metrics, contour agreement assessment and dosimetric fidelity—demonstrate that inherent CBCT image quality characteristics substantially influence the achievable accuracy and reliability of clinical ART implementation.

Contour agreement analysis revealed notable performance differences that corresponded directly to quantitative image quality metrics. Despite comparable spatial resolution between systems (MTF50: 0.36–0.37 lp/mm), Halcyon's superior low‐contrast resolution with 1.65‐fold higher CNR (22.21 vs. 13.42) and 1.78‐fold better HU uniformity (9 vs. 16 HU) resulted in significantly better geometric agreement with pCT‐based reference contours. For the treated breast, Halcyon achieved higher mean accuracy (DSC: 0.92 ± 0.06 vs. 0.87 ± 0.21, *p* < 0.001) with 3.5‐fold lower variability, a pattern that persisted across all evaluated structures where TrueBeam showed 3‐ to 5‐fold higher SD. The superior CNR likely enhanced the AI algorithm's ability to consistently detect subtle soft tissue boundaries, while better HU uniformity reduced artifactual variations that could confound segmentation algorithms. These findings suggest that for automated contouring in low‐contrast anatomies such as breast tissue, CBCT systems with superior low‐contrast resolution provide more reliable inputs for AI‐based segmentation, even when spatial resolution is equivalent. This consistent performance with minimal inter‐patient variability is particularly crucial for automated ART workflows, where predictable segmentation outcomes reduce the need for manual intervention and quality assurance reviews.

Dosimetric analysis revealed paradoxical patterns between target coverage and internal dose metrics that corresponded to the observed HU uniformity and calibration differences. While TrueBeam appeared to maintain better PTV coverage (D_95%_, V_100%_), doses calculated on TrueBeam CBCTs showed significantly increased internal dose metrics (D_mean_, D_2%_). Conversely, Halcyon showed slightly reduced coverage but maintained better overall dose distribution fidelity. This divergent pattern stems from fundamental differences in HU response at water‐equivalent density, where TrueBeam exhibited markedly elevated values compared to both pCT and Halcyon. The HU uniformity difference likely reflects the systems' design characteristics: Halcyon's O‐ring gantry with 154 cm source‐to‐imager distance (SID) provides inherently better scatter management compared to TrueBeam's C‐arm configuration at 150 cm SID.^46^, ^47^ This interpretation is further supported by the cumulative exposure data: despite TrueBeam delivering approximately 5.5‐fold higher cumulative mAs (268.5 vs. 49.1 mAs), and 4.4‐fold higher CTDIvol (4.00 vs. 0.90 mGy), it demonstrated inferior noise characteristics (CNR: 13.42 vs. 22.21; SNR: 94.29 vs. 164.16), indicating that the observed image quality differences are driven by system design rather than exposure levels. Furthermore, differences in reconstruction filter characteristics between the two systems may contribute to the observed noise differences, as kernel selection significantly influences the trade‐off between spatial resolution and noise in reconstructed images. Despite system‐specific calibration curves, these systematic HU deviations propagated through dose calculations, with TrueBeam's reduced uniformity creating offsetting errors that fortuitously aligned at the D95% level while distorting the overall dose distribution. The apparent advantage of TrueBeam in coverage metrics therefore warrants careful interpretation, as the agreement may be attributed to compensating errors rather than accurate dose calculation. Halcyon's superior HU uniformity facilitated more consistent calibration curve application throughout the volume, indicating that its small coverage deviation could represent the true residual uncertainty inherent in CBCT‐based dose calculation rather than systematic error.

Structure‐level mean HU comparison further corroborated these findings (Table 4). Both systems demonstrated systematically positive ΔHU values (deformed pCT − CBCT) across all evaluated structures, indicating that CBCT reported consistently lower raw HU values than the deformed pCT. TrueBeam exhibited substantially larger discrepancies in breast structures (ΔHU: +24.66 vs. +4.64 HU for treated breast). However, the resulting dosimetric impact is not determined by raw HU differences alone, but by how these differences are transformed through each system's HU‐to‐ED calibration curve (Figure 2). In the soft‐tissue density range (ED ≈ 0.94–1.05), the TrueBeam calibration curve diverges substantially from the pCT curve, whereas the Halcyon curve closely tracks the pCT, consistent with the larger dosimetric differences observed for TrueBeam in breast tissue. In the lung density range (ED ≈ 0.2–0.4), all three calibration curves converge, consistent with the smaller between‐system dosimetric differences generally observed for lung structures, although individual dose metrics showed varying patterns that may reflect the sensitivity of dose calculation to tissue heterogeneity at lung‐tissue interfaces.

3D gamma analysis provided spatial validation of the dosimetric discrepancies observed in DVH analysis. While both systems achieved near‐perfect passing rates under standard 3%/3 mm criteria, application of more stringent 1%/2 mm criteria revealed substantial performance differences, with Halcyon maintaining higher passing rates (97.66 ± 1.91%) compared to TrueBeam (90.28 ± 6.51%). To complement the gamma analysis, dose difference analysis was performed to directly quantify dosimetric magnitude differences (Table 7). Halcyon demonstrated smaller absolute dose deviations (mean dose difference: −0.05 ± 0.05 Gy; MAE: 0.20 ± 0.07 Gy) compared to TrueBeam (mean dose difference: −0.26 ± 0.09 Gy; MAE: 0.37 ± 0.08 Gy), with the same trend observed in relative local analysis (Halcyon: −0.84 ± 0.40%; TrueBeam: −1.86 ± 0.47%). The consistency between gamma and dose difference results confirms that the observed dosimetric discrepancies are predominantly attributable to dose magnitude differences arising from HU map variations, rather than spatial displacement of dose distributions. These spatial dose distribution differences were particularly pronounced in heterogeneous regions. For the ipsilateral lung D_2%_, the opposing dose calculation trends (decreased values for Halcyon vs. increased values for TrueBeam) may be related to the measured differences in HU uniformity and HU‐to‐ED calibration between systems. The greater variability in TrueBeam's dose calculations at tissue interfaces is consistent with its reduced HU uniformity identified in phantom measurements. The high inter‐patient variability in TrueBeam's gamma passing rates indicates inconsistent dose calculation reliability across different patient anatomies, highlighting the need to consider system characteristics in ART protocol development.

Our study has several limitations. This analysis focused exclusively on breast cancer patients, and findings may not generalize to other anatomical sites with different tissue compositions and contrast patterns. The patient cohorts between systems differed in clinical characteristics, with the TrueBeam cohort having a higher proportion of patients receiving nodal irradiation (45% vs. 15%) and showing left‐sided predominance (80% vs. 50%), which represent potential confounding factors for between‐system comparisons. Although our within‐system dose comparison design—where the deformed pCT and CBCT for the same patient share identical contours and plan parameters—helps mitigate this concern, the cohort imbalance limits the generalizability of direct between‐system comparisons.

All patients were treated under free breathing conditions, and differences in CBCT acquisition speed between systems, with Halcyon generally providing faster image acquisition than TrueBeam, may have introduced varying degrees of motion‐induced artifacts; future studies incorporating breath‐hold techniques would help isolate the impact of respiratory motion from inherent image quality differences. We evaluated only one AI auto‐segmentation algorithm, and performance characteristics may vary with different algorithmic approaches. The contour agreement analysis relied on deformable registration between pCT and CBCT to establish a common reference frame. Although the same automated FFD algorithm and default settings were applied consistently across both systems without manual intervention, differences in CBCT image quality may influence registration accuracy, which could partially affect the reference contours on the deformed pCT. However, both registration accuracy and segmentation performance are ultimately driven by the same underlying CBCT image quality, and previous studies have reported acceptable DIR accuracy for pCT‐to‐CBCT registration with commercial algorithms.^48^, ^49^

Our dosimetric analysis used forward dose recalculation rather than full plan re‐optimization to isolate the direct impact of CBCT image quality on dose calculation, as re‐optimization could partially compensate for HU‐related inaccuracies and mask the intrinsic effect of image quality differences. However, this approach does not reflect the complete online ART process where plan adaptation is performed. Future studies incorporating full plan re‐optimization would better characterize the achievable plan quality and determine the extent to which the optimizer can mitigate the image quality differences identified in this study. Additionally, the two cohorts used different immobilization devices (breast board for Halcyon vs. delta couch for TrueBeam). However, within each system, all patients were immobilized using the same device and positioning protocol, ensuring consistent setup conditions for within‐system comparisons. The between‐system immobilization differences should nonetheless be considered when interpreting direct between‐system comparisons.

Future studies should extend this analysis to other anatomical sites with greater tissue heterogeneity, such as head‐and‐neck or pelvic regions, and evaluate multiple AI segmentation algorithms to determine whether the observed image quality advantages are generalizable. Additionally, investigations incorporating full plan re‐optimization would better simulate complete online ART workflows, assessing achievable plan quality rather than just forward dose calculation accuracy. Evaluation of next‐generation CBCT platforms with iterative reconstruction capabilities would also be of value, where our framework and baseline data can serve to quantify the impact of improved image quality on contour agreement and dose calculation accuracy within the ART workflow.^50^, ^51^ Overall, these findings emphasize that careful consideration of CBCT image quality characteristics is essential when implementing clinical ART.

## 5 CONCLUSION

These findings demonstrate that quantitative CBCT image quality metrics, particularly CNR and HU uniformity, directly affect both contour agreement and dosimetric concordance in breast ART workflows. Despite comparable spatial resolution (MTF50: 0.36‐0.37 lp/mm), substantial differences in noise characteristics (CNR: 22.21 vs. 13.42; SNR: 164.16 vs. 94.29) and HU uniformity (9 HU vs. 16 HU) between Halcyon and TrueBeam systems translated to measurable differences in AI auto‐segmentation consistency and dose calculation accuracy. Importantly, even when using system‐specific HU‐to‐ED calibration curves, the underlying image quality characteristics fundamentally influenced dosimetric reliability. These results provide quantitative evidence linking CBCT image quality metrics to both contour agreement and dose calculation accuracy within the ART workflow.

## AUTHOR CONTRIBUTIONS

**Sunhwa Kim**: Conceptualization; methodology, validation; writing—original draft. **Young Kyu Lee**: Conceptualization; methodology; review & editing. **Wonjoong Chen**: Conceptualization; methodology; review & editing. **Yunji Seol**: Validation; review & editing. **Byung‐Ock Choi**: Data curation; review & editing. **Young Nam Kang**: Project administration.

## CONFLICT OF INTEREST STATEMENT

The authors declare no conflicts of interest.

## Data Availability

The data supporting the findings of this study are not publicly available due to ethical and legal restrictions related to patient privacy, as the data were derived from anonymized and de‐identified medical records. The data may be made available from the corresponding author upon reasonable request and with appropriate institutional approval.

## 1

See Tables A1 and A2.

**TABLE A1:** HU‐to‐ED table.

Material	Electron density relative to water	Planning CT	Halcyon CBCT	TrueBeam CBCT
Aluminum	2.36	2009.29	1498.18	2236.31
HE cortical bone	1.78	1337.93	1124.15	1734.75
CaC03 50%	1.46	812.85	681.5	1055.32
CaC03 30%	1.27	454.08	395.81	675.46
HE inner bone	1.16	308.6	248.34	539.23
HE liver	1.05	57.16	69.57	217.67
HE brain	1.02	28.17	−20.57	156.83
Water	1.00	−2.7	−34.17	119
HE breast 50:50	0.97	−37.59	−44.48	72.48
HE general adipose	0.94	−66.72	−61.21	11.06
Lung LN‐450	0.44	−503.31	−432.06	−425.14
Lung LN‐300	0.28	−685.53	−608.94	−718.22
Air	0.0012	−1003.73	−1000	−1000

**TABLE A2:** Contour agreement metrics between deformed pCT and CBCT‐based auto‐segmentations.

Machine	Structure	Dice similarity coefficient	Jaccard Index	95% Hausdorff distance (mm)	Mean surface distance (mm)	Volume similarity
**Halcyon** **CBCT**	Contralateral breast	0.91 ± 0.04	0.83 ± 0.07	5.24 ± 1.43	1.92 ± 0.41	0.97 ± 0.02
	Contralateral lung	0.87 ± 0.06	0.78 ± 0.08	3.29 ± 1.44	1.42 ± 0.38	0.99 ± 0.00
	Esophagus	0.72 ± 0.14	0.58 ± 0.15	4.16 ± 2.37	1.66 ± 0.67	0.88 ± 0.10
	Heart	0.95 ± 0.05	0.92 ± 0.08	5.71 ± 3.26	2.44 ± 1.34	0.98 ± 0.05
	Ipsilateral lung	0.91 ± 0.04	0.84 ± 0.07	2.72 ± 0.72	1.24 ± 0.28	0.99 ± 0.00
	Treated breast	0.92 ± 0.06	0.85 ± 0.09	4.87 ± 1.26	1.81 ± 0.55	0.98 ± 0.03
**TrueBeam CBCT**	Contralateral breast	0.83 ± 0.21	0.75 ± 0.20	6.99 ± 2.61	2.36 ± 0.66	0.92 ± 0.22
	Contralateral lung	0.77 ± 0.20	0.65 ± 0.20	3.13 ± 0.46	1.39 ± 0.18	0.93 ± 0.23
	Esophagus	0.71 ± 0.11	0.55 ± 0.12	4.48 ± 2.00	1.74 ± 0.51	0.91 ± 0.06
	Heart	0.90 ± 0.22	0.86 ± 0.23	6.94 ± 3.85	2.55 ± 0.81	0.93 ± 0.23
	Ipsilateral lung	0.81 ± 0.20	0.72 ± 0.19	2.73 ± 0.27	1.24 ± 0.18	0.93 ± 0.23
	Treated breast	0.87 ± 0.21	0.80 ± 0.20	5.72 ± 1.48	2.07 ± 0.40	0.93 ± 0.23
